# A radio-genomics biomarker for precision epidermal growth factor receptor mutation targeting therapy in non-small cell lung cancer

**DOI:** 10.1038/s41598-026-42948-4

**Published:** 2026-03-06

**Authors:** Mitchell Chen, Susan J. Copley, Kristofer Linton-Reid, Patrizia Viola, Yidong Han, Alessio Cortellini, Haonan Lu, Aleksander Mani, Marize Bahket, David J. Pinato, Danielle Power, Andrea G. Rockall, Eric O. Aboagye

**Affiliations:** 1 Department of Surgery and Cancer, Faculty of Medicine, Imperial College London, Hammersmith Hospital Campus, Du Cane Road, London, W12 0NN UK; 2 Imperial College Healthcare NHS Trust, Imperial College Healthcare NHS Trust, Hammersmith Hospital, Du Cane Road, London, W12 0HS UK; 3 North West London Pathology, Charing Cross Hospital, London, W6 8RF UK; 4 Fondazione Policlinico Universitario Campus Bio-Medico, Via Alvaro del Portillo 200, Rome, 00128 Italy; 5 Imperial Centre for Translational and Experimental Medicine (ICTEM), Hammersmith Campus Du Cane Road, London, UK

**Keywords:** Non-small cell lung cancer, Imaging biomarker, Radiogenomics, EGFR mutation, Tyrosine kinase inhibitor, Biomarkers, Cancer, Computational biology and bioinformatics, Genetics, Oncology

## Abstract

Newer-generation tyrosine kinase inhibitors (TKIs) have shown increasing efficacy in cancers driven by specific mutations, with epidermal growth factor receptor (EGFR) alterations remaining the most common actionable targets in non-small cell lung cancer (NSCLC). Treatment decisions are currently guided by tissue sampling and genetic testing, which are limited by procedural risks, patient tolerance, tumour heterogeneity and mutation evolution. Because co-mutations involving EGFR and other targetable genes can diminish treatment response, identifying *exclusive* EGFR mutation, defined by the absence of other actionable alterations, represents a clinically favourable scenario for first-line EGFR-TKI therapy. We developed a CT-based radiomics signature, EGFR-RPV, to predict exclusive EGFR mutational status using NSCLC patients (*n* = 304) from a multi-centre cohort with paired imaging and genomics data, and validated performance in an independent testing set (*n* = 51), alongside transcriptomics enrichment analysis. EGFR-RPV predicted exclusive EGFR mutation with accuracies of 0.77 (95% CI 0.66–0.88) and 0.71 (95% CI 0.54–0.89) in internal and external testing, respectively, and stratified patient prognosis (hazard ratio 2.15, 95% CI 1.50–3.08). FAM190A and CBMO1 were enriched in exclusive EGFR-positive cases, consistent with their roles in cell division regulation and vitamin A biosynthesis, respectively. EGFR-RPV thus offers a non-invasive approach to identify exclusive EGFR mutations, with a potential role in guiding first-line EGFR-TKI use.

## Introduction

Lung cancer is the leading cause of cancer-related deaths worldwide, with non-small cell lung cancer (NSCLC) accounting for 80–85% of its cases^1^. Over 70% of NSCLC cases are diagnosed at advanced stages, carrying poor prognoses^2^. Tyrosine kinase inhibitors (TKI) have excellent response profile in cancers exhibiting certain oncogenic driver mutations, with those relating to epidermal growth factor receptor (EGFR) being the most common targets in NSCLC^3^. Newer generation EGFR-TKIs, such as osimertinib, are more effective against advanced and metastatic NSCLC, with prolonged patient survival, improved quality of life and fewer adverse events compared to chemotherapy^4^, and are now the first line therapy for cancers harbouring suitable mutations^5^.

In precision EGFR-TKI clinical pathways, it is crucial to ensure timely commencement of therapies to maximise patient benefit, avoid unnecessary treatment-related adverse events^6^, and select the best first-line treatment. For example, in the case of immune checkpoint inhibitor (ICI), increased pneumotoxicity has been observed in patients with EGFR mutation^7^. Treatment decision is currently guided by tissue sampling followed by next generation sequencing (NGS)^8^, which is burdened by procedural invasiveness, patient acceptance, tumour heterogeneity and quality of sampled tissue^9^. Up to 30% of patients do not have suitable biopsy sample available^10^. More recently, liquid biopsy with plasma-derived cell free DNA (cfDNA) analysed by digital droplet polymerase chain reaction (ddPCR)⁠ has been proposed as an alternative test^11^, but is limited by confounding effect from non-tumour cfDNA from normal tissue necrosis, lysis of leukocytes after blood collection or clonal haematopoiesis, leading to its suboptimal specificity; and the lack of mutation localisation in multi-focal disease^12^. An overall genotyping error rate of up to 11.1% has been reported for cfDNA^10^. These limitations highlight the need for strategies that can complement existing molecular testing to improve the delivery of precision EGFR-TKI therapy in NSCLC. Imaging-based biomarkers hold notable promise in this regard, given their non-invasiveness, broad availability, and low cost.

Radiomic features are quantitative metrics derived from imaging data and can non-invasively capture important disease information⁠^13–16^. Prior research have demonstrated the utility of radiomics for predicting EGFR mutation, though they have been limited to single mutation prediction^17–20^. In clinical practice, multiple actionable mutations are routinely tested in non-squamous NSCLC^21^. Co-occurring mutations can be found in up to 12.9% of the EGFR mutation positive patients^22^, including up to 3.9% with concomitant anaplastic lymphoma kinase (ALK)^23^ and 1.1% with Kirsten RAt Sarcoma viral oncogene homologue (KRAS) mutations^24^. Co-mutation status is associated with an increased treatment resistance to EGFR-TKI and worse patient survival^22,24–26^. Although radiomics studies have previously investigated the prediction of targetable mutations, including EGFR and KRAS^27–29^, the identification of *exclusive* EGFR mutation, characterised by the absence of other key mutations such as ALK and KRAS, remains underexplored in the literature, despite its clinical relevance for predicting response to EGFR-TKI. In this paper, we introduce a CT-based radiomic biomarker, EGFR-RPV, for the prediction of this important mutation profile.

## Results

### Patient characteristics

We included NSCLC patients presenting to our multi-centre institution between February 2012 and July 2019 (*n* = 304, age (mean ± standard deviation): 67.6 ± 10.7, male: female [M: F] = 174:130) as a discovery cohort, and a dataset^30^ from the Cancer Imaging Archive (TCIA) (*n* = 51, Age: 69.6 ± 8.1, M: F = 35:16) for external testing. The discovery cohort was split 2:1 into training and internal validation sets, balanced for patient’s age, sex, and tumour histology. Exclusive EGFR positivity was defined as the presence of actionable EGFR mutation in the absence of concurrent ALK or KRAS mutations, at the time of patient treatment. 

### Radiomics predictive vector

**Fig. 1:**
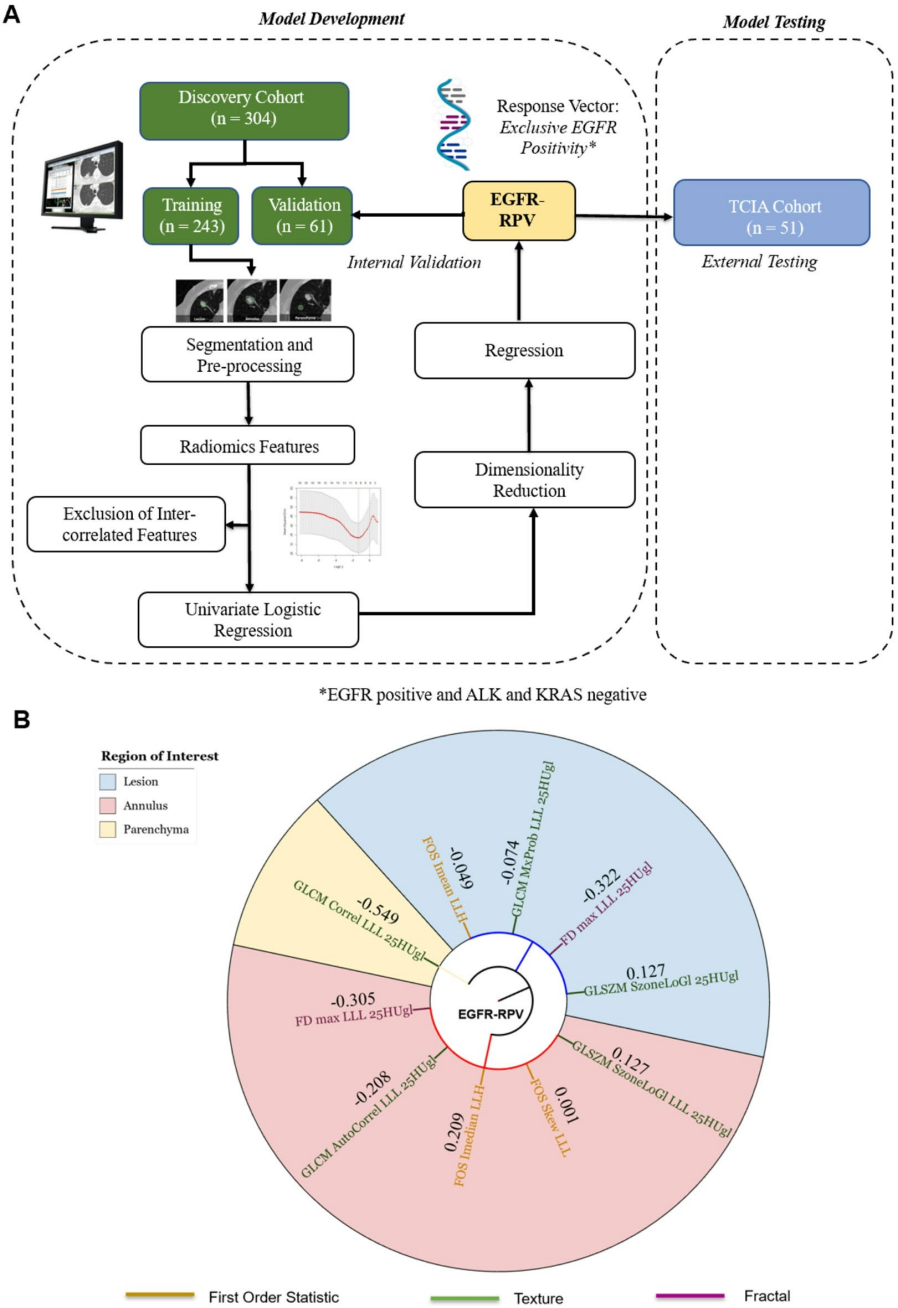
Development and composition of EGFR-RPV. **(A)** EGFR-RPV development and testing pipeline. **(B)** Enriched radiomic features in EGFR-RPV; which were drawn from all three regions of interests (ROIs), with most number of features deriving from the annulus followed by lesion, from all classes including first-order, texture and fractal dimension (FD). Some features are related to wavelet or Laplacian-of-Gaussian (LoG) transformed images.

The biomarker development pipeline is presented in Fig. 1A. EGFR-RPV was developed using multi-regional segmentation, comprehensive radiomic feature extraction in a multi-regional approach (regions of interests (ROI): lesion, perilesional annulus and lung parenchyma), followed by a benchmarking of various dimensionality reduction and regression methods for the best performing statistical learning pipeline.

**Fig. 2:**
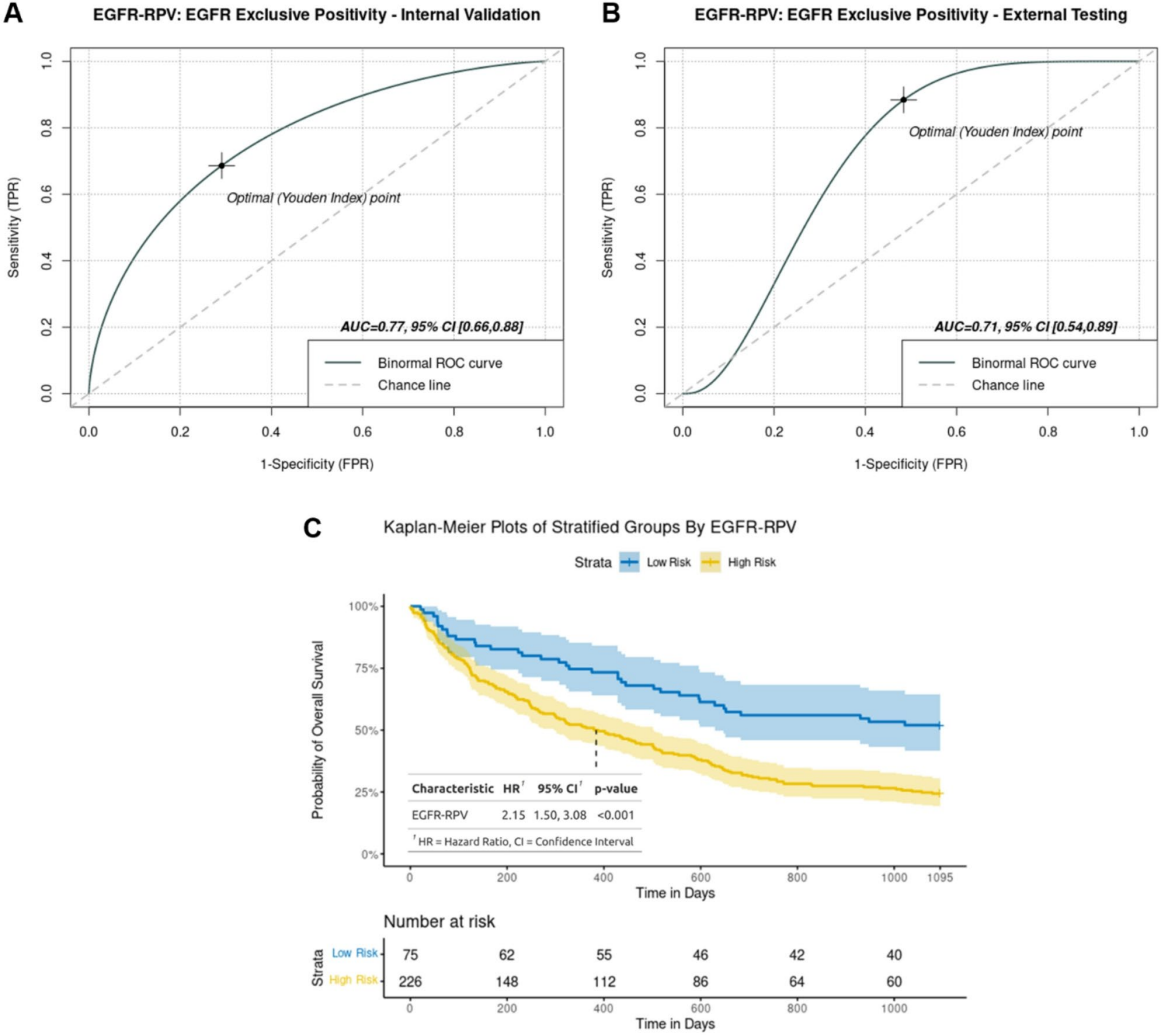
Performance of EGFR-RPV for predicting exclusive EGFR mutation in (**A**) internal validation set. (**B**) external testing set, and (**C**) patient stratification into high and low risk groups in the discovery cohort; three patients with missing survival information were excluded.

EGFR-RPV is a 10-feature composite radiomics vector (Fig. 1B) developed using Spearman and Least Absolute Shrinkage and Selection Operator (LASSO) methods. It predicts exclusive EGFR positivity to an accuracy of 0.77, 95% CI: 0.66–0.88 and 0.71, 95% CI: 0.54–0.89 in the internal and external testing sets, respectively (Fig. 2A and B). The component features belong to first order and higher classes (texture and fractals), extracted from all three ROIs, with the most number (*n* = 5) originating from the peri-lesional area; an observation consistent with the hypothesised distribution of oncogenic cells harbouring driver mutations^31^. EGFR-RPV also delivers effective patient prognostic stratification into high and low risk groups (Cox hazard ratio (HR) 2.15, 95% CI 1.50–3.08, log-ranked *p* < 0.001). (Fig. 2C)

### Logistic regression analysis and clinico-radiomics integration

**Fig. 3:**
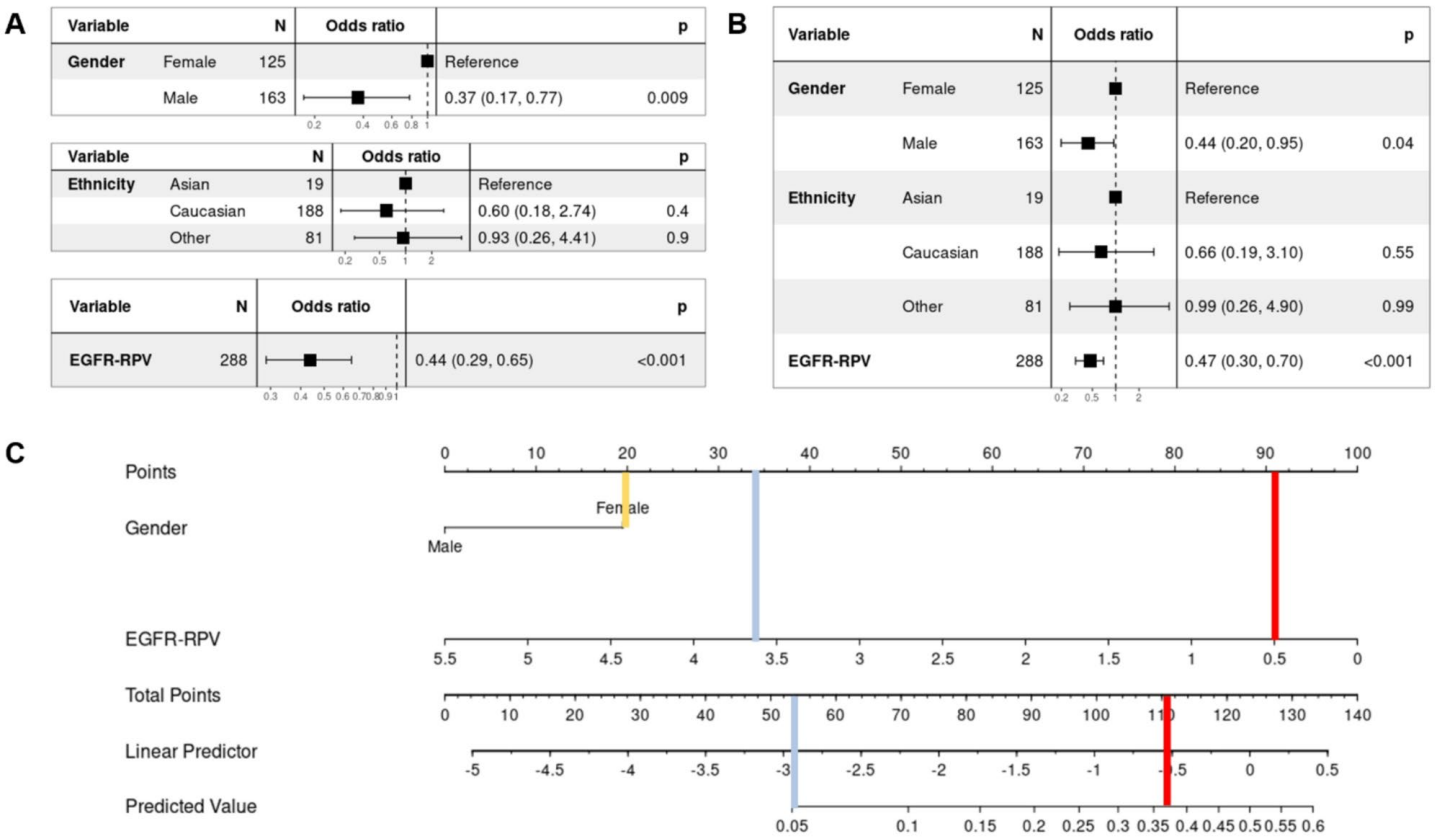
Clinico-radiomics statistical analysis and integration as a clinical-decision nomogram. (**A**) Univariable and (**B**) multivariable logistic regression of clinical features for their clinical predictive values for exclusive EGFR mutation status, showing the statistical significance of patient gender and EGFR-RPV. (**C**) nomogram based on clinco-radiomic features for predicting exclusive EGFR mutation status. The red and blue lines show the calculation of predicted probability of the mutational status in two patients with otherwise identical key clinical characteristics (yellow line), showcasing two use cases.

We have performed univariable and multivariable logistic regression analyses to identify clinical features with statistically significant association with exclusive EGFR mutation status (Fig. 3A and B). The statistical significance of patient sex and EGFR-RPV was established (*p* < 0.05) in both univariable and multivariable analyses. We developed a nomogram using these features with EGFR-RPV to aid in clinical decision making. (Fig. 3C).

### Genomics analysis

Given the wide use of genomics in cancer biomarker research, including their demonstrated utility in various precision oncology scenarios^32,33^ and relevance to radiomics advancements for NSCLC^15,34,35^, we have further performed RNA transcriptomics analysis to advance an understanding of the radio-genomics landscape of NSCLC in the context of exclusive EGFR positivity.

**Fig. 4:**
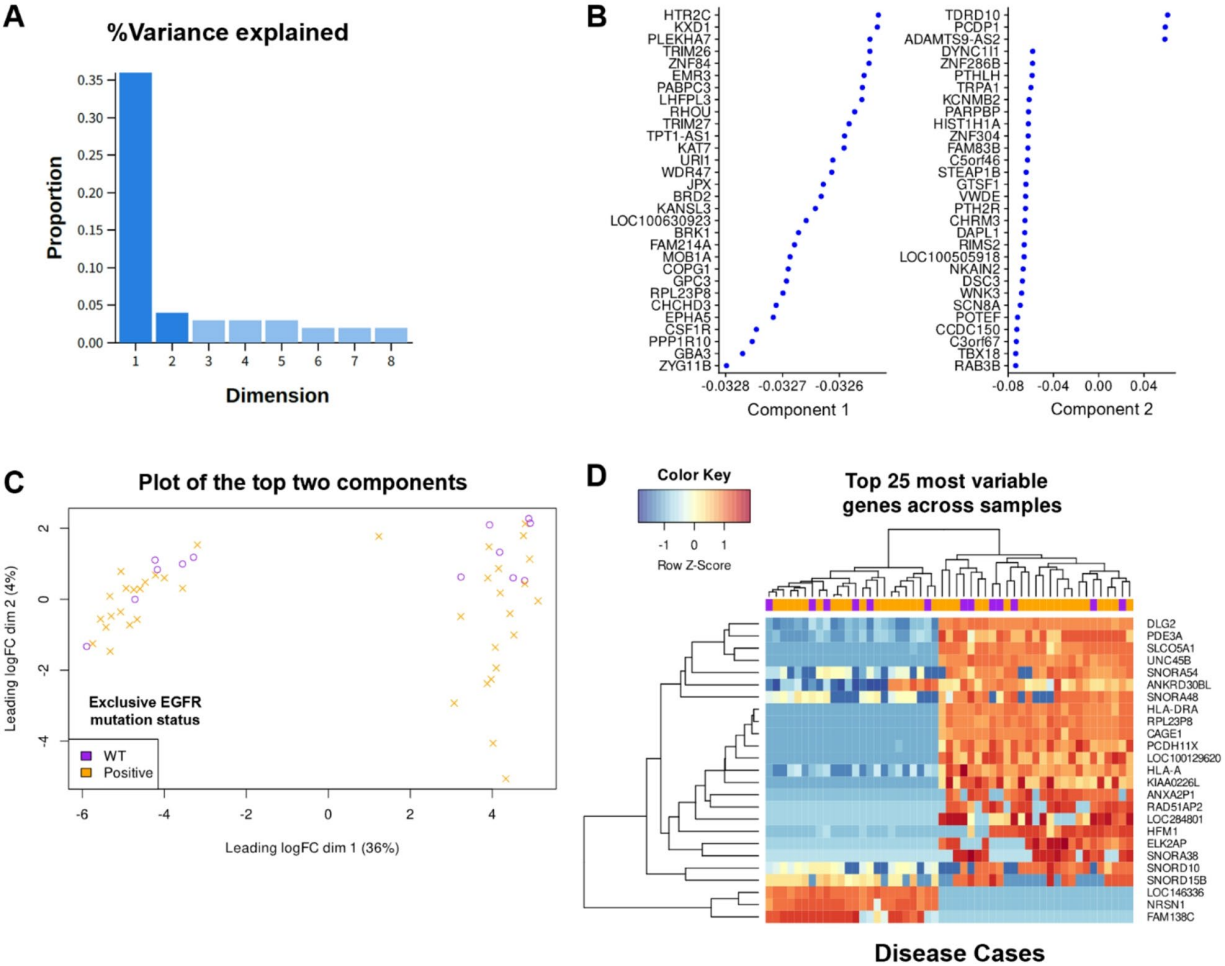
Transcriptomics correlates of exclusive EGFR mutation status. (**A**) Principal component analysis (PCA) of bulk RNA sequencing data showing the first two components accounting for most of the observed variance. (**B**) Top constituent genes of these principal components. (**C**) Unsupervised clustering based on these components do not satisfactorily stratify the patients based on their exclusive EGFR mutation status. (**D**) Hierarchical clustering by Euclidean distance presented as a heatmap again failed to stratify for exclusive EGFR mutation. WT: wild type.

**Fig. 5:**
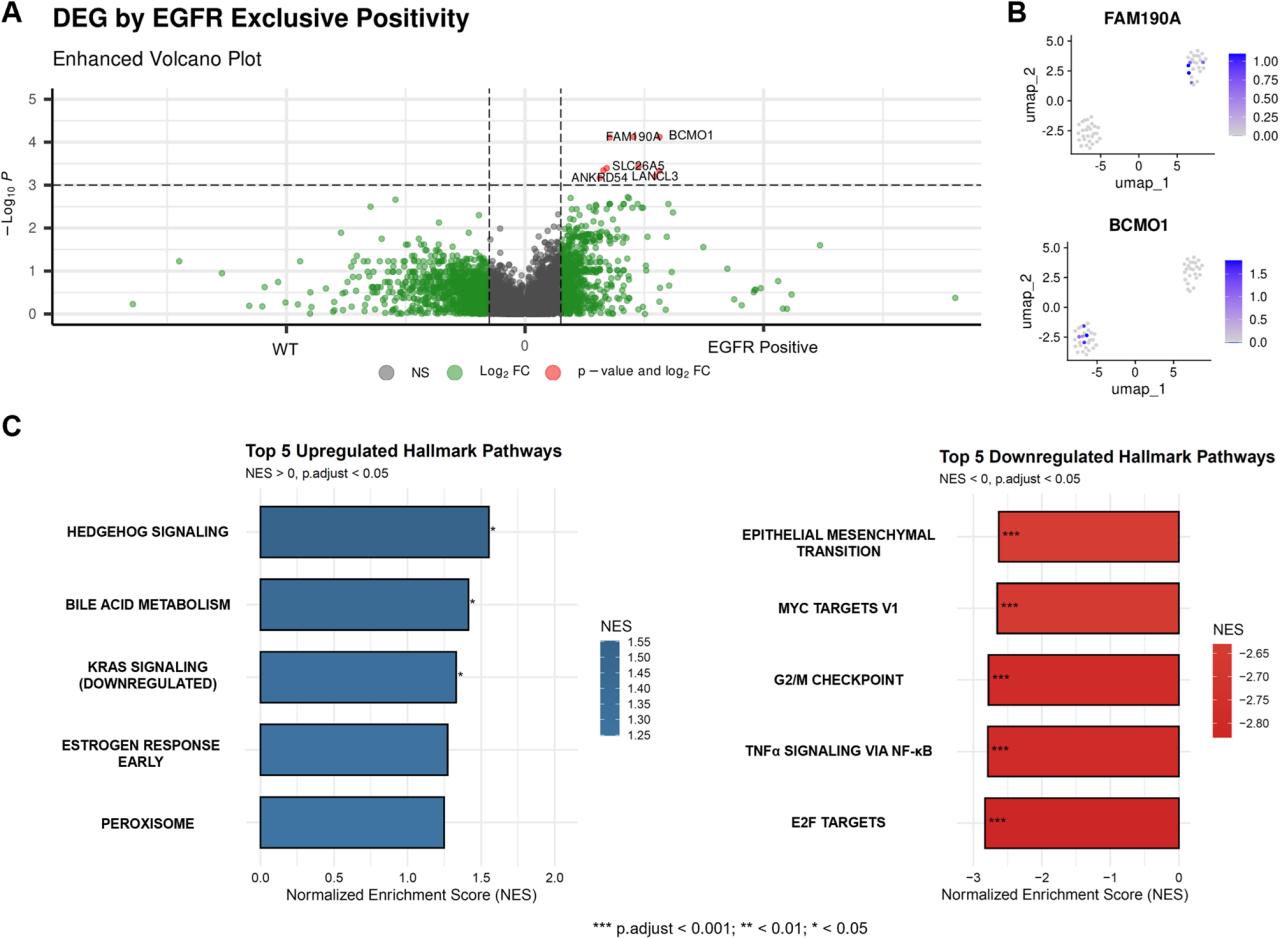
Differential expression gene analysis for exclusive EGFR mutation status. (**A**) Differentially expressed genes (DEG) by exclusive EGFR mutation status. (**B**) Distribution of the top two DEG genes (FAM190A and BCMO1) on Uniform Manifold Approximation and Projection (UMAP). (**C**) Gene set enrichment analysis showing the most upregulated and downregulated pathways in tumours with exclusive EGFR mutation. Note with a cutoff NES of 1.5 for moderate or strong enrichment, only Hedgehog signaling pathway was significantly upregulated. WT: wild type.

In this study arm, we analysed the bulk RNA transcriptomics readouts from the NSCLC Radiogenomics dataset, an independent cohort of 51 patients (Age: 69.6 ± 8.1, M: F = 35:16)^30^. In the latent space formed by the two top ranked principal components which explain most data variance (Fig. 4A and B), we found exclusive EGFR positivity is not distinctly predicted by unsupervised clustering of the samples (Fig. 4C) nor by their hierarchical clustering by Euclidean distance on a heatmap (Fig. 4D). We discovered *FAM190A* and *BCMO1* genes to be most differentially expressed in cases with exclusive EGFR positivity (Fig. 5A), which are expressed in separate clusters on Uniform Manifold Approximation and Projection (UMAP) plot (Fig. 5B). In GSEA, with a normalised enrichment score (NES) cutoff of ± 1.5 to define moderate to strong enrichment, we observed that only the Hedgehog signaling pathway was significantly upregulated in exclusively EGFR mutated samples. In contrast, several pathways were notably downregulated, including epithelial-mesenchymal transition (EMT), MYC targets V1, G2/M checkpoint, TNFα signaling via NF-κB, and E2F targets.

## Discussion

Tissue sampling followed by mutational profiling enables targeted treatment for NSCLC harbouring EGFR mutations, regardless of disease stage. Nevertheless, this approach is burdened by the invasiveness of tissue sampling procedure, tumour heterogeneity and quality of sampled tissue^9^. The more recently introduced liquid biopsy with plasma cfDNA is limited by false positives arising from non-tumour cfDNA, and a lack of mutation localisation in multi-focal disease^12^. To tackle these challenges, we developed a novel, non-invasive, imaging biomarker for guiding clinical decisions using routinely acquired imaging data. It demonstrates good performance for predicting exclusive EGFR mutation and achieves effective patient prognostic stratification.

EGFR mutations are more prevalent in non-smoking, female and East Asian patients^36^. The commonest actionable types of EGFR mutations include del19 (exon 19) and L858R (exon 21), which account for up to 90% of all EGFR mutations in NSCLC^37^. Previously, target mutations such as EGFR, ALK and KRAS were considered mutually exclusive, but their co-mutational status is becoming increasingly recognised for their association with increased treatment resistance to EGFR-TKI and worse patient survival^22,24–26^. This supports a comprehensive analysis of key actionable mutations when determining patient suitability for this treatment.

The definition of exclusive EGFR mutation status in this paper is based on the clinical context and data availability at the time of study. During the study period (2012–2018), KRAS mutation was not considered targetable, as no approved therapies were available for clinical use at that time. The therapeutic landscape changed significantly in 2021, when the first KRAS inhibitor demonstrated clinical efficacy and gained regulatory approval, thereby establishing KRAS as an actionable target^38^. This distinction is crucial for interpreting our results: while KRAS was not clinically targetable during the study period, its presence is now directly targetable and has been associated with reduced benefit from EGFR-TKI^39^. In current clinical practice, the knowledge of the absence of KRAS mutation in EGFR-mutant disease helps to improve treatment strategy by permitting more confident use of EGFR-targeting therapies. Additionally, the definition of exclusive EGFR mutation did not include other currently actionable genes such as ROS1, RET, MET and BRAF V600E^40^.

In precision treatment for NSCLC, the importance of predicting for exclusive EGFR positivity cannot be overstated. First, mutation exclusivity ensures the tumour is primarily dependent on the EGFR-driven pathway, thus maximising the likelihood of a strong therapeutic response to EGFR-TKI. By identifying patients with co-occuringmutations such as ALK and KRAS, we could avoid the use of ineffective or potentially toxic therapies in such cases. In tissue-scarce scenarios, a high predicted probability of exclusive EGFR positivity can justify focused assays while avoiding delays from broad yet low-yield testing; it can also avoid futile therapy in cases where other non-EGFR actionable mutations are present and, in the contemporary setting, redirect such candidates toward more appropriate targeted strategies in subsequent lines. Finally, with their more favourable survival profile, tumours harbouring exclusive EGFR mutations could benefit from more accurate disease prognostication when they are readily identified at the time of diagnosis.

In our study, all patients underwent mutational testing on tissue biopsy specimens, and as such, EGFR-RPV was developed in a cohort where tissue acquisition was feasible. The aim of this study was not to replace tissue- or plasma-based molecular testing, but to evaluate the potential of a rapid, low-cost, non-invasive imaging signature to complement the current diagnostic workflow. When biopsy is performed as standard, EGFR-RPV could provide early molecular insights prior to the availability of biopsy-derived results, and offer a surrogate for longitudinal monitoring without repeated invasive procedures. EGFR-RPV can also provide complementary information in clinical scenarios where standard assays yield inconclusive results, such as when there is inadequate tissue DNA quality due to poor cellularity, necrosis, or degraded FFPE material;^41^ very low variant allele frequency (VAF) in tissue below validated diagnostic thresholds;^42^ or in the case of liquid biopsy, low tumour fraction in circulating cfDNA, particularly in oligometastatic disease or protected compartments (e.g. brain) which can yield false-negative results^43^. Additionally, EGFR-RPV can help to adjudicate discordant tissue versus plasma results, acting as a tie-breaker^44^. In patients with negative or unavailable molecular testing but clinical features strongly suggestive of EGFR-mutant disease, such as those who are female, non-smoker and have adenocarcinoma histology^36^, EGFR-RPV could provide clinical-decision guidance while confirmatory assays are pending. In the above contexts, the utility of EGFR-RPV lies not only in its non-invasiveness, but also in its speed, affordability, and potential to bridge gaps when conventional genomic testing is inconclusive.

To the best of our knowledge, EGFR-RPV is the first imaging biomarker to predict for exclusive EGFR positivity.

Several radiomic biomarkers have previously been presented in literature for EGFR mutation. We searched on PubMed using keywords “CT”, “radiomics”, “EGFR” and “Lung cancer”, for original related radiomics studies published since 2012^13^. We reviewed the bibliometric search results and listed the most relevant ones in Table 2; most were limited to small scale studies, lacked in external validation, and/or suffered from methodological shortfalls such as not meeting the recommendations stipulated by the International Biomarker Standardisation Initiative^45^. None dealt with the question of exclusive EGFR positivity that our study addresses.

Previously, the radiomic features found to be predictive of EGFR mutation were from multiple feature classes. For example, texture features such as gray level size zone (*GLSZM*) and wavelet transformed features have been consistently included in various published radiomic biomarkers for actionable EGFR mutations^18–48^. In our study, we found most constituent features to derive from the peri-lesional ROI; which would be consistent with the hypothesised distribution of oncogenic cells harbouring driver mutations^31^, and supported radiologically on contrast enhanced CT and ¹⁸F-fluorodeoxyglucose-Positron Emission Tomography (FDG-PET) imaging.

Evaluating the specific enriched radiomic features for their biophysical significance, EGFR-RPV includes wavelet transformed first order, texture, FD and wavelet-LoG transformed texture features from the tumour; wavelet transformed first order, texture, FD and wavelet-LoG transformed texture features from the perilesional annulus; and wavelet transformed texture feature from the lung parenchyma, with the most positive weight attached to wavelet transformed first order statistic (*FOS Imedian LLH*) from the perilesional annulus. A raised median intensity of the perilesional area can be associated with tissue invasion into the surrounding lung, as commonly observed radiologically in adenocarcinoma, the most common NSCLC histological subtype to harbour targetable EGFR mutations^49^.

Interestingly, the highest absolute weight is a negative one attached to the only feature from the lung parenchyma (*glcm Correl LLL*), which suggests a negative predictive value the lung parenchyma feature has on the presence of exclusive EGFR mutation. *Glcm* quantifies gray-level zones or the number of connected voxels that share the same gray-level intensity within the image and has been associated with the presence of pulmonary emphysema^50^. This finding would be consistent with the understanding that EGFR mutations are commonly found in adenocarcinomas and non-smokers^49^. With tobacco smoking commonly associated with squamous cell carcinoma of the lung^51^, emphysema-associated radiomic feature could imply squamous cell characteristics not otherwise detected histologically in the sampled tissue, particularly in poorly differentiated or mixed NSCLC histology cases diagnosed on limited or necrotic tissue specimen^52^. This latter hypothesis, if proven, would support the use of radiomics to screen for squamous cell cancer features, which could in turn influence the patient’s initial clinical pathway.

The molecular analysis of paired bulk RNA data provides a complementary genomic perspective that supports the overarching aim of this study through a non-imaging approach, which reflects the central role of genomics in cancer biomarker research, where transcriptomic profiling has demonstrated substantial utility across diverse precision oncology applications^32,33^ and has contributed to key advances in radiomics for NSCLC^34,35^. Incorporating transcriptomic analysis establishes a biologically grounded reference against which our imaging-based innovation can be contextualised. Notably, unlike EGFR-RPV, a genomics-based approach does not readily enable direct prediction of exclusive EGFR mutation. However, the transcriptomic findings offer mechanistic insight into the tumour biology underpinning this molecular subtype. The observed upregulation of *FAM190A* and *BCMO1* in exclusive EGFR-positive cases aligns with their respective roles in regulating aberrant cell division^53^ and catalysing the conversion of β-carotene to vitamin A^54^, the latter being particularly relevant given epidemiological evidence linking β-carotene and vitamin A supplementation to elevated NSCLC risk^55,56^. This genomic analysis therefore enriches the biological interpretability of EGFR-RPV and strengthens the framework supporting its clinical relevance.

The GESA finding of predominantly downregulated pathways shows a selective activation of developmental signaling in tumours harbouring exclusive EGFR mutation, alongside suppression of proliferation- and inflammation-associated pathways. The upregulation of Hedgehog signaling may be associated with tumour stemness and could contribute to the progression of exclusive EGFR-mutant NSCLC ^57^, although such causal relationship would require further validation. The observed downregulation of proliferation-associated pathways (MYC targets, G2/M checkpoint and E2F targets) suggests that such tumours may rely less on canonical proliferation programs, potentially reflecting cell cycle modulation specific to EGFR signaling^58,59^. The suppression of EMT and inflammatory pathway (TNFα signaling viaNF-κB) proposes a downregulation of EMT could correlate with a more epithelial phenotype, consistent with prior observations that EGFR-mutant tumours are less mesenchymal^60^.

The limitations of this study include its retrospective nature and relatively small size of the external testing set. While EGFR-RPV showed promising accuracy in predicting exclusive EGFR mutation status across internal and external validation datasets, further refinements are needed to strengthen its clinical applicability. There are some significant differences between the disease characteristics of the discovery and testing cohorts. For example, in the testing cohort, most patients (82.4%) had early-stage, non-metastatic NSCLC, whereas 41.6% of the discovery cohort presented with stage 3 or 4 disease. Additional statistically significant differences in histological subtype, PD-L1 expression and EGFR exclusive positivity are present between the two cohorts. The observed differences in AUCs, ROC curves, and Youden Index values between the development and testing cohorts likely reflect these dataset differences, which can influence model prediction and the optimal threshold for classification. Despite these differences, however, we note EGFR-RPV maintained good predictive performance across the cohorts, demonstrating good robustness to variations in these characteristics. Nevertheless, further external validation in diverse populations is necessary to confirm the generalisability of EGFR-RPV.

Radiomic features can be affected by the type of scanner, scanning protocol and reconstruction setting used^61^. We have ascertained feature reproducibility by including only reproducible features validated in a test-retest experiment, and features meeting an intraclass correlation coefficient (ICC) threshold. Furthermore, we have used resampling, standardisation, and feature harmonisation techniques, and validated EGFR-RPV in an external testing dataset acquired in a different country (USA) from training (UK), with different scanners and scanning protocols, as well as statistically significant differences in certain cohort characteristics (Table 1). Although the multi-institutional nature of our dataset reduces single-centre bias, residual heterogeneity in CT protocols and hardware could still affect feature stability and model generalisability, which remains an important consideration requiring continued methodological rigour in future works.

**Table 1:** Key radiomics literature on EGFR mutation prediction. Performance metric given in AUROC, unless stated otherwise.

Study	Training	Validation	Performance*	Feature Type	Features	Limitation
Le et al.^48^	TCIA (*n* = 143)	Internal (*n* = 18)	0.778	Hand-crafted features	Wavelet, first order energy	No external
Moreno et al.^49^	TCIA (*n* = 83)	Internal (80:20)	0.857	Hand-crafted and deep learning features	Texture	No external
Wu et al.^46^	Local (*n* = 67)	Cross-validation only	0.882	Hand-crafted features	Shape/surface volume ratio, texture, wavelet features	No external
Zhang et al.^47^	Local (*n* = 297)	Independent dataset (*n* = 127)	0.753	Hand-crafted features	first-order, texture, wavelet features	Only portal venous phase scans routinely performed

Over the past decade, osimertinib has been established as the standard first-line treatment for patients with advanced or metastatic NSCLC harbouring actionable EGFR mutations. However, the field is now shifting: recent landmark trials, such as FLAURA-2^62^ and MARIPOSA^63^, have moved the focus from the development of newer EGFR inhibitors, towards combination strategies aimed at further improving patient outcomes. FLAURA-2 demonstrated improved progression-free survival with the addition of chemotherapy to osimertinib, while MARIPOSA evaluated the combination of amivantamab, a bispecific antibody targeting EGFR and MET, with lazertinib, another third-generation EGFR-TKI. In this context, EGFR-RPV might have a potential role in refining patient selection for such combination approaches. For example, patients with exclusive EGFR-driven disease might represent a subgroup that could derive differential benefit from intensified or combination strategies, pending further investigation.

MET co-alterations have emerged as a clinical challenge in EGFR-mutant NSCLC, with MET amplification recognised as a mechanism of resistance to EGFR-TKI monotherapy^64^. In our study, given its retrospective nature, an evaluation of EGFR-RPV for MET mutation status prediction was not feasible. Although the Ion Torrent Hotspot Panel used in the discovery cohort provided limited coverage of MET hotspot mutations, it did not reliably capture the most relevant alterations, namely MET amplification and exon 14 skipping, and such data was also not provided in the testing cohort. Consequently, MET co-alterations could not be reliably evaluated in this study, prompting future work to assess EGFR-RPV in relation to its prediction of MET–EGFR co-mutational status, to ensure alignment with contemporary molecular oncology practice.

Other future works include testing the biomarker prospectively and evaluating its utility in clinical practice. Additional pertinent outcome measure could include response to treatment. Given the expanding use of EGFR-TKI, we could consider developing a related biomarker for early-stage NSCLC treated with resection followed by adjuvant TKI. In patients with concomitant PD-L1 positivity and EGFR mutation, EGFR-TKI can be combined with ICI for improved systemic therapy; but this is associated with increased incidence of immune-related adverse events; warranting further investigation^65^.

### Methods

#### Data collection

This retrospective study was approved by the institutional review board (IRB) and Health Research Authority UK (HRA: 18HH4616), conducted in accordance with the Declaration of Helsinki, and adhered to the STROBE and REMARK guidelines. Informed consent was obtained from all study participants at the time of the image data acquisition, as in routine clinical practice. The requirement for separate consent for this study was waived by IRB and HRA, due to the study’s retrospective and observational nature and use of de-identified patient data.

The discovery cohort consisted of 304 patients with NSCLC (age (mean ± standard deviation): 67.6 ± 10.7, male: female [M: F] = 174:130) who underwent CT scans and tissue sampling followed by genetic testing at our multi-centre institution between February 2012 and July 2018. An independent cohort of 51 patients (Age: 69.6 ± 8.1, M: F = 35:16) from TCIA was used for external validation^30^. Patients in this cohort have had mutational tests for actionable mutations, and additionally bulk RNA sequencing of their tumour tissue specimen using a HiSeq 2500 (Illumina, San Diego, USA) system.

**Fig. 6:**
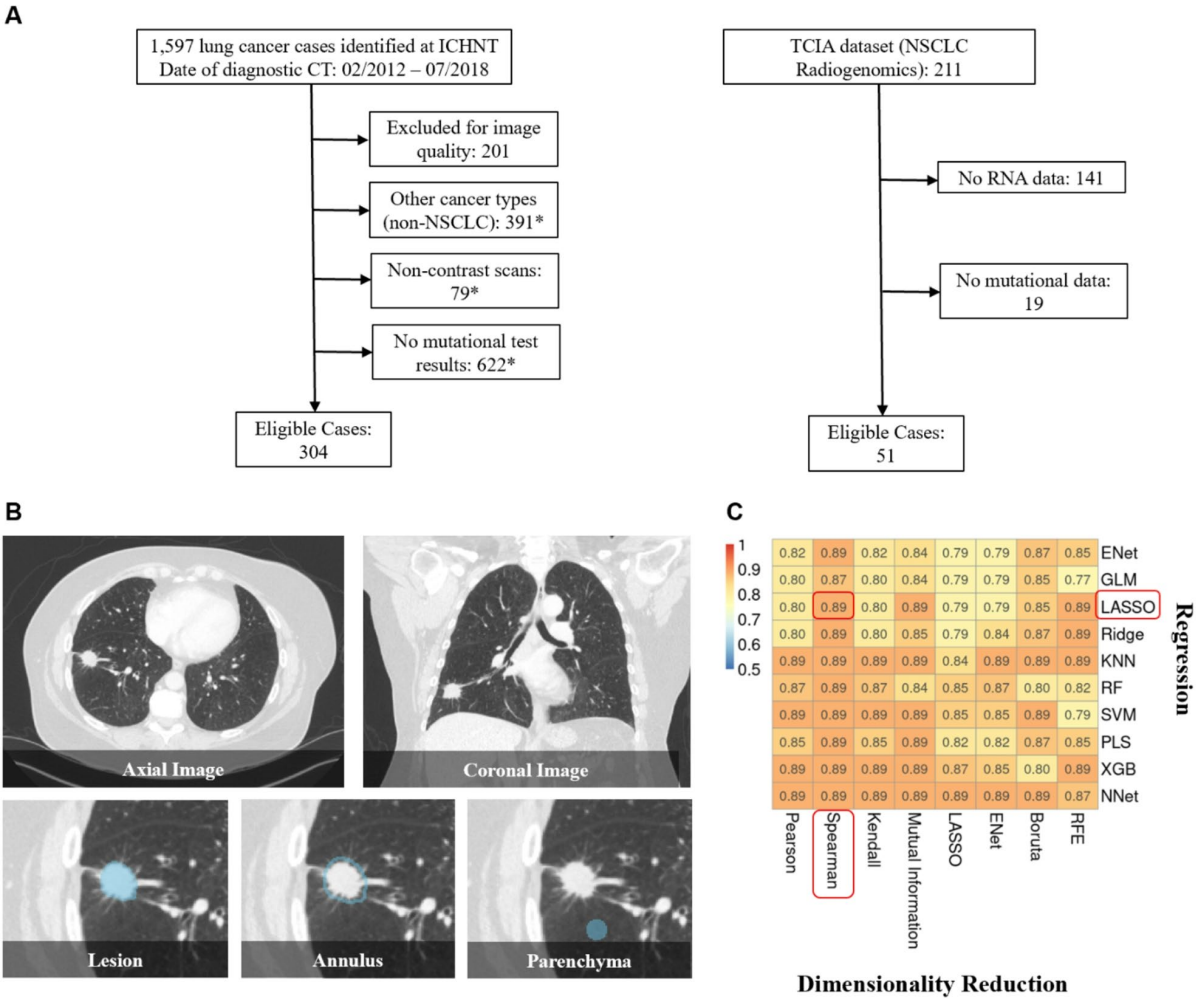
Study methodology. (**A**) Flow diagrams of the discovery and external testing cohort. (**B**) Multi-label segmentation for radiomics feature extraction. (**C**) Optimal dimensionality reduction and regression method selection, as assessed by their area under the receiver operating curve. In our case, the combination of LASSO and Spearman yielded the best performance and was adopted for use. ENet: elastic net; GLM: generalised linear model; LASSO: least absolute shrinkage and selection operation; KNN: k-nearest neighbour, RF: random forest; SVM: support vector machine; PLS: partial least squares, XGB: extreme gradient boosting; NNet: neural net; RFE: recursive feature elimination.

Clinical data including patient demographics and tumor histology were collected from electronic patient record. Actionable EGFR mutation was the primary study endpoint. Patient overall survival was documented up to 3 years post-diagnosis^66^. It is defined as the time from the baseline diagnostic CT to 3-year follow-up or patient death of any cause, whichever occurred earlier. We excluded cases with tumor histology other than NSCLC, missing clinical or molecular data, or with non-contrast or thick axial slice (> 3 mm) scans. Patient flow diagrams and characteristics are presented in Fig. 6A, and Table 2, respectively.

**Table 2:** Characteristics of patients included in the study and p-values showing statistical differences between the discovery and testing cohorts.

Age (years)	Discovery Cohort (*n* = 304) No. (%)	Testing Cohort (*n* = 51) No. (%)	*p*-value Training vs. Testing
Median Standard Deviation Range	67.6 10.7 32–92	69.6 8.1 50–85	0.2085
**Sex**			
Female Male	130 (42.8) 174 (57.2)	16 (31.3) 35 (68.7)	0.1268
**ECOG Performance Status**			
0 1 2 3 4 Unknown	148 (48.7) 91 (30.0) 43 (14.1) 19 (6.3) 2 (0.7) 1 (0.3)		
**T Stage**			
1 2 3 4 Unknown	94 (30.9) 84 (27.6) 47 (15.4) 77 (25.) 2 (0.7)	21 (41.2) 21 (41.2) 5 (9.8) 4 (7.8) 0	< 0.05*
**N Stage**			
0 1 2 3 Unknown	118 (38.8) 44 (14.5) 78 (25.6) 63 (20.7) 1 (0.3)	41 (80.4) 5 (9.8) 5 (9.8) 0 0	< 0.05*
**Metastases**			
0 1 Unknown	158 (52.0) 145 (47.7) 1 (0.3)	48 (94.1) 3 (5.9) 0	< 0.05*
**Histological type**			
Adenocarcinoma Other	286 (94.1) 18 (5.9)	45 (88.2) 6 (11.8)	< 0.05*
**PD-L1 expression**			
TPS < 1% TPS ≥ 1%	252 (82.9) 52 (17.1)	28 (54.9) 23 (45.1)	< 0.05*
***EGFR*** **mutation**			
Negative Positive **Exclusive Positivity**	262 (86.2) 42 (13.8) 34 (11.2)	38 (74.5) 13 (25.5) 12 (23.5)	0.142 < 0.05*

Note: p-values were calculated using Wilcoxon rank-sum test for continuous variables, and chi-square test for categorical variables. Percentage figures are given in brackets, unless otherwise specified. ECOG: Eastern Cooperative Oncology Group; disease stage based on International Association for the Study of Lung Cancer (IASLC) 7th edition; TPS: tumour proportion score; Exclusive EGFR mutation is defined as where it is positive in the absence of ALK and KRAS positivity. *denotes statistically significant difference.

### Genetic testing

In the discovery cohort, DNA was extracted from formalin fixed paraffin embedded (FFPE) tissue using the Qiagen QI Symphony DSP DNA Minikit (Qiagen N.V., Hilden, Germany)^67^. The mutational screening was performed by next-generation sequencing using the Ion Torrent Hotspot Panel (Ion Torrent Systems, now part of Thermo Fisher Scientific, South San Francisco, CA, USA). The assay comprised 207 amplicons in 50 oncogenes frequently mutated in solid tumours, including EGFR, KRAS, NRAS, BRAF, and PIK3CA. Reference Sequences NM_005228.3, NM_004985.3, NM_002524.4, NM_004333.4, and NM_006218.2 were used to screen the EGFR, KRAS, NRAS, BRAF and PIK3CA genes, respectively. Fluorescence in situ hybridisation (FISH) was used in parallel for ALK rearrangements (EML4-ALK translocation).

In the testing cohort, EGFR, KRAS, and ALK mutation status were available. Single-nucleotide variant detection was performed using the SNaPshot assay (Applied Biosystems, now Thermo Fisher Scientific, Waltham, MA, USA)^30^. EGFR mutations were assessed in exons 18–21, KRAS mutations in exon 2. ALK rearrangements were evaluated using FISH for detection of EML4–ALK translocations.

We defined exclusive EGFR positivity as EGFR positivity in the absence of ALK and KRAS mutation; actionable EGFR, ALK, and KRAS mutations, at the time of writing, which were covered in both cohorts, were considered.

### Image acquisition

All patients in the discovery cohort had contrast-enhanced chest CTs demonstrating a primary NSCLC at the time of diagnosis. The three centers (A, B, C) at our institution used different scanners (Site A: Siemens Definition AS+; Site B: Philips Brilliance and Philips Ingenuity; Site C: Siemens Definition AS+) and institutional scanning protocols with a peak kilovoltage (kVp) ranging from 100-140kVp (120kVp), tube current 120–650 mA (mean 200 mA) and slice thickness 0.625–3 mm (median: 1.5 mm), and contrast given in the portal venous phase. Scans were acquired with subjects in supine position with arms at sides, from the apex of the lungs to the adrenal glands within a single breath-hold.

Patients in the testing cohort received their CT scans at Stanford USA^30^, performed using various scanners with 80–140 kVp (mean 120 kVp), 124–699 mA (mean 220 mA) and slice thickness of 0.625–3 mm (median: 1.5 mm). Contrast enhancement phase, subject positioning, breath holding and scan coverage were similar to those in the discovery cohort.

### Multi-label segmentation

Two chest radiologists, blinded to clinical and histological data, with 13 and 9 years of experience, doubly reviewed all scans using both mediastinal (width, 350 HU; level 40 HU) and lung (width, 1500 HU; level, -600 HU) window settings, and performed semi-automated segmentation of the tumour, peri-lesional annulus of 5 mm thickness, and a spherical parenchymal patch of 2 cm diameter in the same or an ipsilateral pulmonary lobe, where there is no appreciable aerated lung remaining. This multi-region approach (Fig. 6B) is currently the mainstay in lung cancer radiomics workflow^68^. All delineations were made using 3DSlicer (Slicer Community, Boston, USA)^69^.

### Image processing and radiomic features extraction

After segmentation, the imaging data were resampled to a uniform voxel size of 1 × 1 × 2 mm and analysed for a total of 1,998 radiomic features from each scan (666 features per ROI), using an in-house software (TexLab 2.0), in Matlab 2020b (MathWorks Inc., Natick, MA, USA)^15,70^.

The computed features included ones pertaining to tumour image intensity, shape and texture from the original, wavelet and Laplacian of Gaussian (LoG) transformed images, which are compliant with Image Biomarker Standardisation Initiative (IBSI)^45,71^. We have additionally extracted a texture descriptor not yet covered by IBSI, fractal dimension (FD), to capture complex spatial patterns not well characterised by traditional metrics^15,72^. This was captured using a box-counting algorithm, which involved overlaying grids of varying box sizes over the ROIs and computing the number of boxes required $N\left(\epsilon\right)$ to cover the object as a function of box size $\epsilon$. The fractal dimension was then estimated as the negative slope of the linear regression line fitted to the log-log plot of $\mathrm{l}\mathrm{o}\mathrm{g}\left(N\left(\epsilon\right)\right)$ versus $\mathrm{l}\mathrm{o}\mathrm{g}(1/\epsilon)$.

The computed radiomic features were standardised to a mean of zero and standard deviation (SD) of one. To further countering batch effects resulting from inter-scanner and inter-site variabilities, features were harmonised using ComBat^73^, in keeping with IBSI recommendation^45,71^.

Inter-observer radiomic feature reproducibility was assessed by calculating ICC on the basis of a two-way random model. There were 1,452 features found to have an ICC greater than or equal to 0.8, thus deemed reproducible and included in subsequent dimensionality reduction and regression steps. Test-retest repeatability was assessed using a cutoff ICC of greater or equal to 0.9, based on the publicly available RIDER dataset (*n* = 29), where repeated CT scans were taken 15 min apart for every participant^74^.

### Model development and validation

Common dimensionality reduction and regression methods were benchmarked to select the best combination for achieving optimal performance, as assessed by predictive performance in the internal validation cohort (Fig. 6C). In this work, the combination of Spearman and Least Absolute Shrinkage and Selection Operator (LASSO) yielded the best performance and was therefore adopted to develop the predictive biomarker, EGFR-RPV. This study has a radiomics quality score of 23/36^75^.

We have performed univariable and multivariable logistic regression analyses to identify clinical features with statistically significant association with exclusive EGFR mutation status and developed a nomogram based on EGFR-RPV and clinical features deemed statistically significant in these analyses, using R package *rms.*

### Statistical analysis

All statistical analyses and machine learning were performed using R version 4.3.0 (R Project for Statistical Computing, http://www.r-project.org/). The statistical tests were two-sided, with a p-value significance threshold of 5% adopted throughout. Differences between cohorts were tested using the analysis of variance test for continuous variables and the chi-square test for categorical variables. Kaplan-Meier plots were used to evaluate the utility of the model for patient prognostication, and log-rank test used to assess survival curve differences. Receiver operating characteristics (ROC) analysis was used to assess the predictive performance of EGFR-RPV. Univariable and multivariable regression models were used to investigate the effects of EGFR-RPV and various clinical features on exclusive EGFR mutation.

### Genomics analysis

Bulk RNA reads were aligned to the human genome (hg19) using the alignment algorithm STAR version 2.3 with 91 bases of splice junction overhangs. Next, the readouts were normalised on an individual gene basis. We computed counts per million (CPM) using R package *edgeR* and retained readouts with a threshold of CPM of 0.5 then *log2* transformed and performed hierarchical clustering with heatmap based on Euclidean distance. We found the feature latent space using principal component analysis (PCA), followed by Uniform Manifold Approximation and Projection (UMAP) and differential expression analysis to identify the most differentially expressed genes based on exclusive EGFR mutation status. For GSEA, Hallmark gene sets from the MSigDB database were obtained using R package *msigdbr*^76^. Pre-ranked GSEA was conducted using R package *clusterProfiler*, to identify the most significantly enriched pathways associated with exclusive EGFR mutation status, which are ranked by their NES.

## Data Availability

Our institutional study data (clinical, genetics and imaging) are retrospective in nature and protected through institutional compliance; and can be shared as per specific institutional review board (IRB) requirements. Upon reasonable request, a data sharing agreement can be initiated between the interested parties and the clinical institution following institution-specific guidelines. The gene expression dataset analysed during the study are from the NSCLC Radiogenomics public domain dataset, available in the Gene Expression Omnibus (GEO) repository, accessed via accession number GSE103584. Its paired imaging and clinical data are deposited in The Cancer Imaging Archive (TCIA), which can be accessed through the NSCLC Radiogenomics collection at: (https://www.cancerimagingarchive.net/collection/nsclc-radiogenomics).

## Code availability

All R scripts and guidance for use can be found on GitHub: https://github.com/scat2801/egfr-rpv/.
